# Nutritional evaluation and growth of infants in a Rwandan neonatal follow‐up clinic

**DOI:** 10.1111/mcn.13026

**Published:** 2020-06-11

**Authors:** Jessica Bradford, Kathryn Beck, Alphonse Nshimyiryo, Kim Wilson, Christine Mutaganzwa, Silas Havugarurema, Patient Ngamije, Alphonsine Uwamahoro, Catherine M. Kirk

**Affiliations:** ^1^ Faculty of Global Health Delivery University of Global Health Equity Kigali Rwanda; ^2^ Boston Children's Hospital Boston Massachusetts USA; ^3^ Kirehe District Hospital Ministry of Health of Rwanda Kirehe Rwanda

**Keywords:** early growth, growth monitoring, malnutrition, preterm infants, primary health care, sick and small newborns, undernutrition, quality of care

## Abstract

Children born preterm, low birth weight (LBW) or with other perinatal risk factors are at high‐risk of malnutrition. Regular growth monitoring and early intervention are essential to promote optimal feeding and growth; however, monitoring growth in preterm infants can be complex. This study evaluated growth monitoring of infants under 6 months enrolled in Paediatric Development Clinics (PDCs) in rural Rwanda. We reviewed electronic medical records (EMR) of infants enrolled in PDCs before age 2 months with their first visit between January 2015 and December 2016 and followed them until age 6 months. Nurse classification of anthropometric measures and nutritional status were extracted from the EMR. Interval growth and length‐for‐age, weight‐for‐length, and weight‐for‐age z‐scores were calculated using World Health Organization anthropometry software as a ‘gold standard’ comparison to nurse classifications. Two hundred and ninety‐four patients enrolled and had 2,033 visits during the study period. Referral reasons included prematurity/LBW (73.8%) and hypoxic ischemic encephalopathy (28.2%). Nurses assessed interval growth at 58.7% of visits, length‐for‐age at 66.4%, weight‐for‐length at 65.6% and weight‐for‐age at 66.4%. Nurses and gold standard assessment agreed on interval growth at 53.3% of visits and length‐for‐age at 63.7%, weight‐for‐length at 78.2% and weight‐for‐age at 66.3%. At 6 months, 46.5% were stunted, 19.9% were wasted and 44.2% were underweight. There were significant challenges to optimizing growth and growth monitoring among high‐risk infants served by PDCs, including incomplete and inaccurate assessments. Developing tools for clinician decision support in assessing growth and providing specialized nutritional counselling are essential to supporting optimal outcomes in this population.

**Key messages**

- There is a lack of guidance on appropriate mechanisms for monitoring and managing malnutrition in infants under 6 months of age, particularly among children with perinatal risk factors such as prematurity, low birth weight or neonatal encephalopathy, which may require specialized guidelines for low‐resource settings.
- Despite training and mentorship, nurses were unable to correctly and consistently monitor growth among high‐risk infants in this context, including challenges in the correct plotting of anthropometrics using standard World Health Organization growth charts.
- Children born preterm, low birth weight or with other perinatal risk factors showed high rates of malnutrition and early growth failure even when enrolled in a clinic designed especially for their routine nutritional follow‐up and intervention. This early growth failure contributes to the burden of wasting, stunting and underweight in older children.
- The follow‐up care of preterm infants and other infants with perinatal risk requires greater attention for feasibility in low‐resource settings and specialized tools to aid healthcare workers in growth monitoring and early identification of growth failure are needed.

## 1 INTRODUCTION

Globally, under‐five mortality has decreased substantially over the past few decades as countries worked towards Millennium Development Goals (MDGs) and now the Sustainable Development Goals (SDGs; You et al., 2015). Although decreases in neonatal mortality have lagged behind improvements in under‐five mortality, more and more high‐risk infants are surviving the neonatal period. It is well known that infants born with perinatal complications due to prematurity, low birth weight (LBW), hypoxic ischemic encephalopathy (HIE) or other problems may have ongoing challenges requiring follow‐up. These infants are at‐risk for developmental delays, poor nutrition, increased mortality during childhood and non‐communicable diseases later in life (Lawn et al., 2014). However, in resource‐limited settings, few programmes exist to meet the ongoing developmental, nutritional and healthcare needs of infants born premature, LBW, with HIE, or with congenital problems, increasing the risk of morbidity and mortality.

Infants born with perinatal complications are at increased risk for developing undernutrition, in both the acute and chronic forms (Christian et al., 2013; Danaei et al., 2016). Research among these populations of children in sub‐Saharan Africa has shown high rates of stunting, wasting and underweight that are often at least double national prevalence (Kirk et al., 2017; Tann et al., 2018; Van den Boogaard et al., 2017). Although optimal weight gain in healthy, term infants has been studied and defined, there are no currently accepted international standards of how to monitor growth in premature infants, with a variety of methods used, including grams per day, grams per kilogram per day and z‐scores (Fenton et al., 2017). Additionally, there is no single currently accepted standard growth chart for use in infants born prematurely. World Health Organization (WHO) standard growth charts require use of corrected age when used for preterm infants. There are also a variety of other charts specific for preterm infants; however, they are normed on different populations (Villar et al., 2018). In all cases, the use of these charts well relies on accurate gestational age, which is a major limitation in low‐ and middle‐income countries. The tools and assessments for monitoring growth in infants <6 months present many challenges, particularly for infants born premature and LBW infants. Routine monitoring of growth in infants <6 months is left out of many national screening programmes (Lopriore, Dop, Solal‐Celigny, & Lagnado, 2007) despite evidence of malnutrition among children in this age range (Kerac et al., 2011). It was only within the past 6 years that the WHO made specific recommendations in regard to severe acute malnutrition (SAM) for infants <6 months [World Health Organization (WHO), 2013].

Rwanda has made tremendous progress in its achievement of MDG4, reducing under‐five mortality from 152 deaths per 1,000 live births in 2005 to 50 deaths per 1,000 live births in 2015 (National Institute of Statistics, 2015). There have been improvements in neonatal mortality as well during this period, in part due to the expansion of inpatient care for sick and small newborns (Hansen et al., 2015). In 2014, Rwanda Ministry of Health, UNICEF, and Partners In Health/Inshuti Mu Buzima (PIH/IMB) collaborated to develop and implement the Paediatric Development Clinics (PDCs) to provide a medical home for health, nutritional and developmental care of these vulnerable infants and children following hospital discharge from neonatal units. A description of the PDCs was previously published (Ngabireyimana et al., 2017). PDCs are currently operating at two district hospitals in the Eastern province of Rwanda and eight health centres in those districts. Reasons for referral to PDC include infants with prematurity (gestational age at birth <37 weeks), LBW (birth weight less than 2,000 g), HIE, cleft lip or cleft palate, as well as children with hydrocephalus, trisomy 21 or other suspected chromosomal disorder, children identified with developmental delay, and children under 12 months of age discharged from the hospital after management of SAM. A nurse and social worker trained on the PDC protocol conduct assessments of patients in the PDCs, with general practitioner support as needed. During the visits, infants are screened for danger signs, have anthropometric measures taken to determine their nutritional status, receive a developmental assessment and have other clinical conditions evaluated according to the diagnosis at referral to PDC.

During the nutritional assessment, for infants <6 months, nurses check the infant's weight, length and head circumference, calculate the interval growth from the prior visit and plot weight‐for‐age (WFA), length‐for‐age (LFA) and weight‐for‐length (WFL) z‐scores using WHO child growth standards (WHO, 2006) on a WHO growth chart, using the corrected age for infants born prematurely with a known gestational age at birth. Referral to inpatient care or nutritional counselling is then provided to caregivers according to the results of the nutritional assessment and whether the child has medical complications associated with malnutrition or not.

This study aimed to investigate the quality of nutritional evaluations of infants <6 months in the PDCs as well as the nutritional status of infants enrolled in the PDCs <6 months.

## 2 METHODS

### 2.1 Study setting

We conducted this study in six PDC sites: Rwinkwavu District Hospital (DH), Cyarubare Health Centre (HC), Kabarondo HC, Ndego HC, Ruramira HC and Kirehe DH. These PDCs were located in the Rwinkwavu DH and Kirehe DH catchment areas in Kayonza and Kirehe Districts, respectively, in rural Eastern province of Rwanda. Rwinkwavu DH and Kirehe DH supervise 24 HCs serving a catchment area of over 600,000 people (National Institute of Statistics, 2012) plus more than 50,000 people in Mahama Refugee Camp with 2 additional HCs. These health facilities were all managed by the Rwanda Ministry of Health and received PIH/IMB support since 2005. As part of the introduction of the PDC, all nurses in the PDC received a 5‐day training on the PDC protocol, which covered taking anthropometrics, plotting growth and identifying poor growth. In addition, mentorship was provided by PDC nurse mentors from the district hospital to health centres and mentorship of hospital PDC nurses by PIH/IMB.

### 2.2 Study design and population

This was a descriptive retrospective cohort study on the nutritional status of infants under 6 months enrolled in PDC. We included in our analysis patients who were enrolled in PDCs between January 1, 2015 and December 31, 2016 with age less than 2 months at enrolment and who remained in care through 6 months of age (age at each PDC visit adjusted for prematurity days when gestational age was <37 weeks). The PDC follow‐up period included visits that occurred from January 1, 2015 through September 30, 2017. All infants who met these inclusion criteria were included in this study, regardless of the diagnostic criteria that led to the infant's enrolment in PDC.

### 2.3 Data collection and definition of variables

#### 2.3.1 Data collection

Data on socio‐economic and clinical characteristics and anthropometric measurements recorded at PDC enrolment and each visit were extracted from the PDC's routine electronic medical record (EMR) for analysis. The EMR is an OpenMRS (Seebregts et al., 2009) system that stores data for the PDCs. PDC data are first recorded on paper forms at every visit before a team of permanent trained data collectors enter those data into EMR within 1 week of a visit.

#### 2.3.2 Definition of variables

‘Small for gestational age (SGA)’ was defined as birth weight < 10th percentile for gestational age using the INTERGROWTH‐21st preterm growth charts (Villar et al., 2014Re).

‘Age at each PDC visit/chronological age’ was calculated by taking the date of visit minus the child's date of birth.

‘Corrected age’ was defined as a premature (born <37 weeks gestational age) infant's chronological age minus the number of weeks born early, where weeks born early is defined as 40 weeks minus gestational age.

‘Stunting’, ‘underweight’ and ‘wasting’ were defined as LFA z‐score < −2, WFA z‐score < −2 and WFL z‐score < −2 based on WHO growth charts, respectively (WHO, 2006). Severity of undernutrition was categorized as moderate if the z‐score was <−2 and ≥−3 and as severe if the z‐score was <−3.

‘Interval growth’ at each PDC visit was calculated by taking the child's weight at the most recent visit and subtracting the weight at the previous visit from it and dividing by the number of days in between those two weights to have an average weight gain in grams per day. Adequate interval growth for infants 0–3 months is 20 g/day and 15 g/day for infants from 3 to 6 months (Ministry of Health, 2017).

‘Gold standard assessments’ were defined as using Stata software to calculate interval growth, z‐scores and corrected age based on date of birth and date of visit, as well as documented anthropometric measurements on each PDC visit.

### 2.4 Data analysis

We used descriptive statistics to summarize data on socio‐demographic and clinical characteristics of infants using frequencies and percentages for categorical data and median and interquartile ranges for continuous data. The 2006 WHO Child Growth Standards (WHO, 2006) were used to calculate z‐scores at each PDC visit. The interval growth and proportions of infants who were stunted, underweight and wasted at 3 and 6 months of age were calculated based on anthropometric measurements (height and weight) recorded on a visit date closest to 3 or 6 months of age. The data were analysed using Stata v.15.1 (Stata Corp, College Station, TX, USA).

### 2.5 Ethical considerations

This study received ethical approvals from the Rwanda National Ethics Committee and the Ministry of Health.

## 3 RESULTS

A total of 294 infants meeting inclusion criteria were enrolled in the study, of which 51.4% were female. Among those with known gestational age at birth (*n* = 208; 70.7%), 63.9% were born with gestational age less than 37 weeks; 29.3% of children had no documented gestational age. Of those with known birth weight, 57.9% were born with birth weight less than 2.0 kg; 5.4% of children had no documented birth weight on file. Among 193 infants with both known gestational age and birth weight, 32.7% of infants enrolled were SGA. The most common reason for referral was prematurity or LBW (73.8%), followed by HIE (28.2%). There were 5.4% infants referred for both prematurity or LBW and HIE. The median age at enrolment was 0.4 months. Half of the infants (50.0%) were enrolled at one of the district hospital PDCs, with the remainder of patients followed at health centre PDCs. Half of the infants received infant formula (54.1%) and half of mothers received a food package (46.6%) at some point in the infant's first 6 months as part of their treatment. The demographic characteristics of the patients and caregivers can be seen in Table 1.

**TABLE 1:** Patients' socio‐demographic and clinical characteristics

Characteristic	*n*	%
**Child characteristics**		
Child's sex		
Male	143	48.6
Female	151	51.4
Child's gestational age (*n* = 208)		
≥37 weeks	75	36.1
<37 weeks	133	63.9
Child's weight at birth (*n* = 278)		
≥2,500 g	77	27.7
2,000–2,499 g	40	14.4
1,500–1999 g	116	41.7
1,000–1,499 g	44	15.8
<1,000 g	1	0.4
Small for gestational age (SGA; *n* = 193)		
No	97	50.3
Yes	96	49.7
**Reasons for referral**		
Prematurity/low birthweight (LBW)		
No	77	26.2
Yes	217	73.8
Hypoxic ischemic encephalopathy (HIE)		
No	211	71.8
Yes	83	28.2
Other conditions^a^		
No	274	93.2
Yes	20	6.8
Multiple conditions		
No	268	91.2
Yes	26	8.8
Paediatric Development Clinic (PDC) of enrolment		
Rwinkwavu DH	97	33.0
Cyarubare HC	29	9.9
Kabarondo HC	46	15.7
Ndego HC	42	14.3
Ruramira HC	30	10.2
Kirehe DH	50	17.0
Child's age (in months) at enrolment in PDC, median [IQR]	0.4	[−0.5–0.8]
Number of PDC visits by 6 months of age, median [IQR]	6	[5–8]
**Household and caregiver characteristics**		
Caregiver's level of education (*n* = 140)		
No education	20	14.3
No formal education level completed	64	45.7
Primary school completed	49	35.0
Secondary or higher completed	7	5.0
Years of school completed by caregiver, median [IQR], *n* = 140	5	[3–6]
Number of children in household, median [IQR], *n* = 128	2	[1–4]
District of residence		
Kayonza	239	81.3
Kirehe	54	18.4
Ngoma	1	0.3
**Nutritional support**		
Ever received maternal food package		
No	157	53.4
Yes	137	46.6
Ever received formula		
No	135	45.9
Yes	159	54.1
Ever received both maternal food package and formula		
No	242	82.3
Yes	52	17.7

*Note.* *n* = 294 unless otherwise specified.

Abbreviations: DH, district hospital; HC, health centre; IQR, interquartile range.

a Other conditions include central nervous system infection, trisomy 21, severe malnutrition under 12 months, hydrocephalus, cleft lip or palate, or other developmental delays.

Table 2 presents nutritional assessments completed at each PDC visit among the total 2,044 PDC visits during the study period. Clinic staff calculated an interval growth at 58.7% of visits during the study period, and recorded LFA at 66.4%, WFL at 65.6% and WFA at 66.4% of visits. The infant's age was corrected for gestational age at birth 23.9% of the time among infants with a recorded gestational age and a gestational age < 37 weeks.

**TABLE 2:** Completeness of assessments of child nutritional status at every visit

Nutrition indicator	Number of PDC visits (*N* = 2,033)	%
Interval growth calculated by nurse		
No	839	41.3
Yes	1,194	58.7
Length for age z‐score calculated by nurse		
No	682	33.6
Yes	1,351	66.4
Weight for length z‐score calculated by nurse		
No	699	34.4
Yes	1,334	65.6
Weight for age z‐score calculated by nurse		
No	683	33.6
Yes	1,350	66.4
Corrected age calculated by nurse (*n* = 1,071)		
No	815	76.1
Yes	256	23.9

Abbreviation: PDC, Paediatric Development Clinic.

Agreement between the nurse assessment and gold standard calculations is shown in Table 3. There was 53.3% agreement between nurses' assessment and the gold standard for interval growth, 63.7% for LFA, 78.2% for WFL and 66.3% for WFA. There was agreement in nurse calculation of corrected age and gold standard calculated corrected age in 14.8% of the visits among infants with a recorded gestational age and a gestational age < 37 weeks with data on both calculations.

**TABLE 3:** Level of agreement between gold standard and nurse calculations of nutrition indicators

Nutrition indicator	*n* ^a^	%
Interval growth calculated by Stata and nurse (*n* = 1,183)		
Not matching	552	46.7
Matching (or difference < 1)	631	53.3
Length for age z‐score calculated by Stata and nurse (*n* = 1,246)		
Not matching	452	36.3
Matching	794	63.7
Weight for length z‐score calculated by Stata and nurse (*n* = 1,222)		
Not matching	266	21.8
Matching	956	78.2
Weight for age z‐score calculated by Stata and nurse (*n* = 1,255)		
Not matching	423	33.7
Matching	832	66.3
Corrected age calculated by Stata and nurse (*n* = 256)		
Not matching	218	85.2
Matching	38	14.8

a *n* = Number of Paediatric Development Clinic (PDC) visits where we have valid data on both the nurse and Stata calculation.

Nutritional status of children at 3 and 6 months of age is displayed in Table 4. The gold standard nutritional assessment for all infants at 3 months showed that 79.1% had appropriate interval growth from 0 to 3 months, 51.8% were not stunted, 86.7% were not wasted and 57.4% were not underweight. At 6 months, 45.8% of infants had appropriate interval growth from 3 to 6 months, 53.5% were not stunted, 80.1% were not wasted, and 55.8% were not underweight. Among only preterm/LBW infants aged 3 months, 79.5% had appropriate interval growth from 0 to 3 months, 44.6% were not stunted, 86.8% were not wasted and 46.3% were not underweight. At 6 months, 43.9% of preterm/LBW infants had appropriate interval growth from 3 to 6 months, 45.1% were not stunted, 77.9% were not wasted and 47.2% were not underweight.

**TABLE 4:** Infants' nutritional status at 3 and 6 months of age

Variable	At 3 months of age						At 6 months of age					
	All		Preterm or LBW infants		Infants with HIE		All		Preterm or LBW infants		Infants with HIE	
	*n*	%	*n*	%	*n*	%	*n*	%	*n*	%	*n*	%
Interval growth												
Adequate^a^	219	79.1	163	79.5	67	83.75	132	45.8	94	43.9	38	47.5
Inadequate	58	20.9	42	20.5	13	16.25	156	54.2	120	56.1	42	52.5
Stunting												
Normal	148	51.8	95	44.6	55	67.9	153	53.5	97	45.1	57	74.0
Moderate	55	19.2	42	19.7	16	19.8	69	24.1	62	28.8	12	15.6
Severe	83	29.0	76	35.7	10	12.4	64	22.4	56	26.1	8	10.4
Wasting												
Normal	248	86.7	186	86.9	70	88.6	230	80.1	166	77.9	68	86.1
Moderate	26	9.1	21	9.8	6	7.6	43	15.0	36	16.9	8	10.1
Severe	12	4.2	7	3.3	3	3.8	14	4.9	11	5.2	3	3.8
Underweight												
Normal	167	57.4	100	46.3	69	84.2	160	55.8	101	47.2	59	74.7
Moderate	51	17.5	49	22.7	5	6.1	61	21.2	54	25.2	10	12.7
Severe	73	25.1	31	31.0	8	9.8	66	23.0	59	27.6	10	12.7

Abbreviations: HIE, hypoxic ischemic encephalopathy; LBW, low birth weight.

a Adequate interval growth defined as ≥20 g/day for 0–3 months or ≥15 g/day for 3–6 months of age.

## 4 DISCUSSION

**FIGURE 1:**
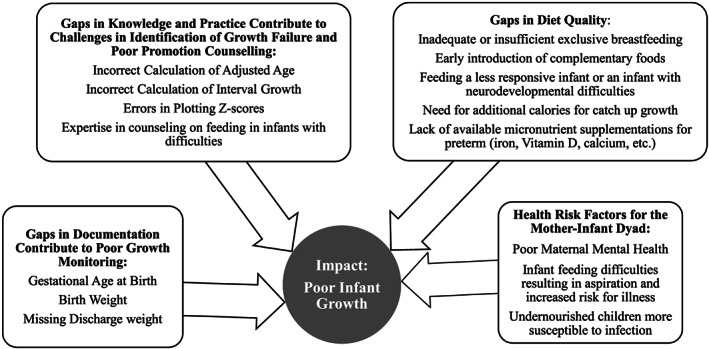
Factors that contribute to poor growth in infants enrolled in the Paediatric Development Clinic (PDC)

Our study highlights a number of challenges in monitoring growth and nutritional status in this population of high‐risk infants, despite training and mentorship of nurses in this specific aspect of care. There were gaps in completeness of assessment at PDC visits, in documentation of key patient history information and in accuracy of nutritional assessments compared with the gold standard. Rates of malnutrition in this population were high particularly at 6 months of age, at which time the global recommendation is to transition infants from exclusive breastfeeding to complementary feeding (see Figure 1).

Interval growth and z‐scores were not documented by nurses in one third or more visits. This incomplete documentation of assessments is of concern because the primary aim of the PDC is to be able to identify growth and other challenges early for appropriate intervention. By not completing the full assessments, early signs of growth failure may be missed. Lack of completed nutrition assessments and documentation were also gaps observed in national nutrition programs implemented in Brazil and Bangladesh (Abud & Gaíva, 2015; Saha et al., 2015). This is also a common barrier identified in the implementation of the Integrated Management of Childhood Illness (IMCI) where nutritional assessments are completed in as few as a quarter of visits (Billah et al., 2017; Horwood et al., 2009). A study of well‐baby visits in South Africa found that only half of children's nutritional status was classified (Sokhela, Sibiya, & Gwele, 2018). In addition, records from maternity and neonatal units are often incomplete, with high numbers of patients lacking a known gestational age at birth or having missing information regarding the birth weight, both of which are critical for appropriate growth monitoring in preterm infants. This reflects challenges at a number of levels. Late or inadequate antenatal care can result in inability to assess gestational age, which is a common challenge in LMICs (Lee, Blencowe, & Lawn, 2018). In addition, by having limited accuracy of data on gestational age, it is often not possible to determine SGA from LBW and appropriate for gestational age; however, their growth trajectories are different (Bocca‐Tjeertes, Bos, Kerstjens, de Winter, & Reijneveld, 2014). Documentation in health facilities may miss key information, such as birth weight, and exchange of information between health facilities may result in missing data when children are transferred for follow‐up care. The majority of the patients enrolled in the PDCs are referred from hospital neonatal units. However, the patients could have been born at home, at the health centre, or at the hospital, with each referral to the next level requiring accurate documentation and transfer of gestational age and birth weight.

Additionally, we saw large discrepancies between nurse calculations and gold standard calculations. This may be explained by several challenges. Calculation of the interval growth in an infant requires several pieces of information and basic arithmetic to assess, including current weight, previous weight and number of days between the two weights. Calendars are not always available to determine number of days between visits, and calculators for computation are sometimes lacking. The lack of accuracy in assessment of children's growth trajectory—particularly for small babies—was also observed in a study in Nigeria, (Ezeofor, Garcia, Ibeziako, Mutoro, & Wright, 2016) and overall poor accuracy of growth monitoring was seen in Zimbabwe and Ghana (Laar, Marquis, Lartey, & Gray‐Donald, 2018; Marume, Mafaune, Maradzika, & January, 2017). This has important implications for the quality of care delivery and likely contributes to missed opportunities for early intervention for signs of growth failure. To address this in the PDC, we are developing an mHealth application that can calculate these parameters for nurses, and so they only have to focus on accuracy of anthropometric measures.

In addition to challenges with the way that growth is monitored, our study showed high numbers of children who are not meeting growth targets, even very early in infancy. Nearly half of infants were stunted and underweight at 6 months, and 20% were wasted, and both wasting and underweight increased from the rates observed at 3 months of age. The highest rates of malnutrition were seen among infants born preterm or LBW. The nutritional vulnerability of infants <6 months is unique in that they rely often exclusively on the mother to provide all of their nutritional and fluid needs through breastfeeding; the nutritional changes that take place in the first 6 months (mechanical, physiological, biochemical and protective) are very rapid, more than changes during other periods of life (Lucas & Zlotkin, 2003). Among those born LBW, these differences may have even greater impact on their nutritional status (Kerac et al., 2010). We were not able to determine from our data why there was an in increase in wasting from 3 to 6 months. Exclusive breastfeeding rates are high in Rwanda, with 87% of infants under 6 months reported to be exclusively breastfed (National Institute of Statistics, 2015). However, the percentage of those exclusively breastfed does decrease from 0 to 6 months, with 94% of infants 0–1 months, 90% of infants 2–3 months and 81% of infants 4–5 months exclusively breastfed (National Institute of Statistics, 2015) indicating potential early introduction of complementary foods. In our study, a large proportion of infants had received infant formula due to failure to breastfeed successfully. This is likely due to a combination of factors including inadequate establishment of breastfeeding while inpatient (de Silva et al., 2019) and difficulty of PDC providers to problem solve once an infant had difficulties with breastfeeding (Beck et al., 2018). In addition to missing out on many benefits of breastfeeding, use of infant formula exposes children to additional risks in contexts such as rural Rwanda where access to safe water is a challenge (Anttila‐Hughes, Fernald, Gertler, Krause, & Wydick, 2018). Additionally, the transition to complementary foods, which should take place at 6 months, is a nutritionally vulnerable time when breastmilk alone cannot meet infant's nutritional needs. In Rwanda, only 58% of infants 6–8 months are fed complementary foods (National Institute of Statistics, 2015). With the high rates of malnutrition in PDC patients already observed when they reach 6 months, this vulnerable period of transitioning complementary feeding will require additional support and specialized interventions for children with feeding difficulties to prevent further growth failure (Kerac et al., 2014). Although we are aware of the declining rates of breastfeeding in Rwanda as the child ages during the first 6 months, these data are not routinely captured in the PDC so we do not know the extent to which this impacts our population. However, early introduction of complementary foods could be contributing to increasing rates of malnutrition we observed. Additionally, despite global recognition that preterm infants require additional micronutrient supplementation (World Health Organization, 2011), only iron is available. Other key micronutrients including Vitamin D and calcium are not available for infant supplementation. More detailed information on feeding practices and feeding issues are needed in this PDC population to better identify their contribution to poor growth.

Knowledge about nutritional status of infants <6 months is lacking, but particularly among infants born with medical vulnerabilities. Although data on malnutrition under 6 months are not routinely tracked, a multicounty assessment found a median prevalence of acute malnutrition of 15% based on WHO growth standards for children under 6 months (Kerac et al., 2011). The high rates of malnutrition during toddler years that is seen among infants born preterm and LBW in other studies (Kirk et al., 2017; Van den Boogaard et al., 2017) may be related to nutrition and growth challenges that begin at an early age, as we have identified in this study. Additionally, this study supports prior studies that infants born SGA continue to lag behind those born appropriate for gestational age in terms of nutritional outcomes. For these infants, continued nutritional support is important, but prevention is essential for infants to reach their optimum growth potential. However, the prevention of malnutrition is reliant on early identification of growth failure, quality growth monitoring and appropriate health and nutrition interventions that encompass the mother‐infant dyad. Poor maternal mental health can contribute to child undernutrition (Ruel et al., 2013) and neurological impairments can lead to feeding difficulties and increased risk of aspiration and subsequent illness and early mortality (Kerac et al., 2014; Olusanya & Nair, 2019). The Community Management of At‐risk Mothers and Infants (C‐MAMI) tool provides health workers with assessment, intervention and management guidance for at risk mothers and infants under 6 months who are nutritionally vulnerable and fills a gap in available tools for infants under 6 months (Emergency Nutrition Network (ENN) et al., 2018). The C‐MAMI tool was recently adapted for use in the PDCs to address gaps in the quality of growth monitoring (Beck et al., 2018).

This study has some limitations to note. We relied on data available within the EMR system, and so missing data could overestimate the lack of growth monitoring occurring during visits. In addition, the gold standard assessments were conducted post hoc using measurements taken and documented by the nurse during clinic visits. The accuracy of measuring height or taking weight was not assessed in this study; however, the observed differences in nurse classification of poor growth and gold standard using the same input measurements still provide very valuable information about the quality of growth monitoring in neonatal follow‐up.

## 5 CONCLUSION

This study shows ongoing issues early in infancy with poor growth among infants with medical vulnerabilities following discharge from neonatal units, despite being enrolled in PDCs. It highlights the need for improvements in early nutritional support among these patients, and strategies to improve quality of care delivery in the PDC around nutrition are ongoing. Adequate recognition of poor growth is needed for problems to be addressed. In addition, strategies to prevent prematurity, intrauterine growth restriction, and feeding difficulties that may arise in the first days of life for preterm infants are needed alongside interventions like the PDC to provide essential early intervention and follow‐up care for high risk infants. Further research is also needed to help develop more appropriate tools and global guidance for the assessment of growth in infants born prematurely.

## CONFLICTS OF INTEREST

The authors declare that they have no conflicts of interest.

## CONTRIBUTIONS

JB led the study design, data interpretation and drafted the manuscript. KB, KW and CMK contributed to study design, data interpretation and manuscript writing. AN analysed the data, drafted and reviewed the manuscript. CM, OB, SH, PN and AU contributed to acquisition of data and critically reviewed the manuscript.
